# Intraoperative surprise evidence of bronchial rent during lung surgery: a case report

**DOI:** 10.11604/pamj.2022.42.255.33790

**Published:** 2022-08-08

**Authors:** Ravi Damodhar Nikhade, Charuta Pravin Gadkari, Aishwarya Santosh Pingley

**Affiliations:** 1 Narendra Kumar Prasadrao (NKP) Salve Institute of Medical Sciences and Research Centre and Lata Mangeshkar Hospital, Nagpur, India

**Keywords:** Tuberculosis, spinal, management, case report

## Abstract

Among multiple causes of tracheobronchial rent, most common is iatrogenic factor. Whenever there is surprise evidence of bronchial wall tear while doing lung surgery, tracheal tube extubation and postoperative management pose a challenge. We report a 16-year-old girl, weighing 27kg, a case of pulmonary Koch's who presented with hydropneumothorax on left side. She had a prolonged course on mechanical ventilation, was gradually weaned off and extubated in intensive care unit (ICU) with implantable cardioverter defibrillator (ICD) in-situ. However, chest X-ray continued to show loss of bronchovascular markings and high-resolution computed tomography (HRCT) thorax revealed multiple cavitatory lesions, hydropneumothorax from upper to lower lobe, ground glass opacities on left side and mediastinal shift towards right side. Hence, she was posted for left lung decortication. Decortication was done using one lung ventilation protocol with 28 Fr left sided double-lumen endobronchial tube (DLT). While checking for leaks before closure, it was noted that exhaled tidal volume was unacceptably low and a rent on left main bronchus of around 2x2 cm with scarred borders was detected. The rent was repaired with tissue patch suturing by the surgeons. After the procedure, DLT was exchanged with endotracheal tube (ETT) no 6. Patient was managed with elective ventilation post-operatively in ICU for 48 hours and extubated uneventfully. A vigilant monitoring of vital parameters and close communication with surgeons is important for detecting and managing any perioperative complication during lung surgery. Elective ventilation could play a significant role for healing a big rent in trachea-bronchial area.

## Introduction

Tracheobronchial tear is defined as partial or complete laceration or puncture in the tracheobronchial tree secondary to a blunt or penetrating trauma or due to iatrogenic interventions. Tracheobronchial tear occurring intraoperatively can result while doing dissection during chest surgery or while intubating the trachea. Management includes determining the severity and extent of the tear, protecting the airway and adopting adequate ventilation strategies. This poses a challenge because the gas exchange is inadequate, hemodynamics are unstable and repairing the tear is difficult from an anatomical point of view [1]. Timely diagnosis, prompt ventilatory intervention and management remain crucial to good recovery.

## Patient and observation

**Figure 1:**
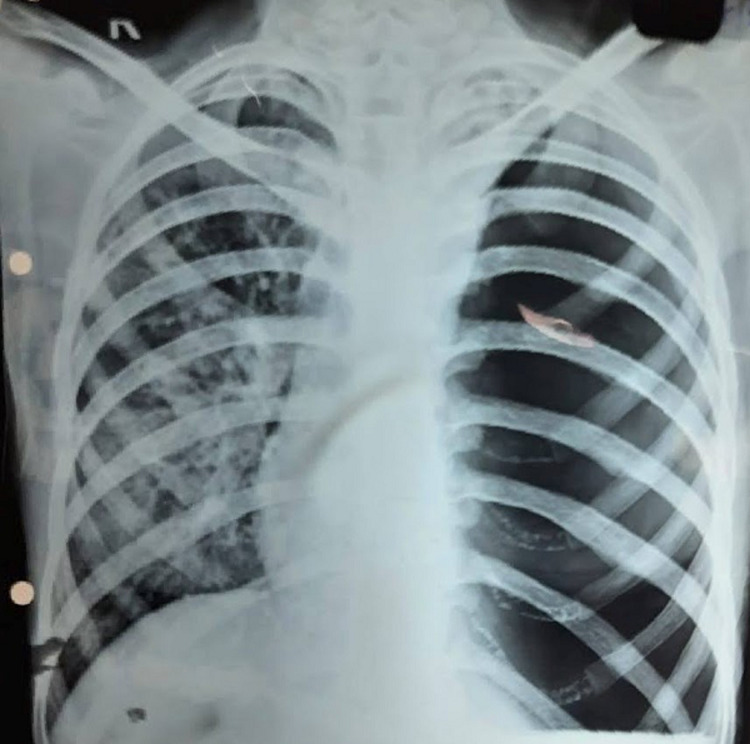
chest X-ray showing tension pneumothorax on left side

**Patient information:** a 16-year-old female patient weighing 27 kg, known case of pulmonary Koch´s on AKT presented to casualty with respiratory distress and hypotension. Chest X-ray showed tension pneumothorax with mediastinal shift towards right side (Figure 1). Emergency ICD insertion and endotracheal intubation was successfully conducted. Left-sided ICD was inserted between 7^th^ and 8^th^ rib in mid axillary line. Patient had an event of desaturation while on mechanical ventilation on 5^th^ day which was managed with repositioning of ICD and titrating ventilator parameters. She was weaned off gradually and extubated with ICD in-situ. Eventually patient developed left sided hydropneumothorax and HRCT showed multiple cavitatory lesions, hydropneumothorax from upper to lower lobe, ground glass opacities and mediastinal shift towards right side, hence she was posted for left lung decortication surgery. Preoperative assessment, routine hematology investigations were done. Written consent was obtained, patient was taken in operating room and standard monitors were attached.

**Clinical findings:** the plan of anaesthesia was thoracic epidural anaesthesia followed by general anaesthesia. However, since patient was not comfortable with placement of epidural catheter before general anesthesia (GA), it was deferred at this point of time. The patient was sedated with injection midazolam and injection fentanyl, intravenous induction of general anesthesia was done with injection lignocaine, injection propofol and injection succinylcholine was used to facilitate endotracheal intubation. Direct laryngoscopy was done (CormackLehane grade 1) and airway was secured with a styleted 28 French left-sided double-lumen endotracheal tube (DLT), which was inserted uneventfully. Auscultation was done to confirm appropriate placement of the DLT. After the patient was positioned in right lateral decubitus for the procedure, auscultation of chest revealed lung isolation was achieved to the optimum. After initiating single lung ventilation, surgeons performed left lung decortication in the usual fashion.

**Diagnostic assessment:** on conclusion of surgery, the left lung was re-inflated to check for any leak as is practiced for any thoracic surgery. However, the exhaled tidal volume was unacceptably low and a significant air leak was observed. Further evaluation revealed an annular tear located along the lateral wall of the left mainstem bronchus. The damage was observed to be far from the tip of the DLT.

**Therapeutic intervention:** left lung was isolated again and defect was repaired with a tissue patch followed by pleural flap. After the repair was completed, there was no evidence of air leak and closure of the chest was done in the conventional manner. The patient was shifted to the supine position. Under direct laryngoscopy, the DLT was exchanged with a single lumen endotracheal tube no 6.

**Follow up and outcome:** extubation plan was discussed with surgeons who speculated that if patient developed even single bout of vigorous cough during extubation, there was a chance of disruption of repair, hence it was collectively decided to electively ventilate the patient for next 48 hours in order to gain some strength over sutures of repaired area. Patient was ventilated with lung protective ventilation in pressure regulated volume control mode (PRVC) with: a) FiO2 0.5; b) tidal volume 6ml/kg; c) positive end-expiratory pressure (PEEP) of 5cm H_2_O (establishing a peak pressure of less than 20cm H_2_O); d) a respiratory rate of 16 per min.

Appropriate post-operative pain relief was provided by administering continuous fentanyl infusion. The patient was electively ventilated in pediatric intensive care unit (PICU) with pressure regulated volume guaranteed (PRVC) mode for next 48 hours till maximum peak pressure of 20 cm H_2_O. Eventually, good respiratory effort and satisfactory tidal volumes were confirmed. Extubation was done without any complications on the third postoperative day and patient was discharged from the hospital after 7 days.

## Discussion

**Figure 2:**
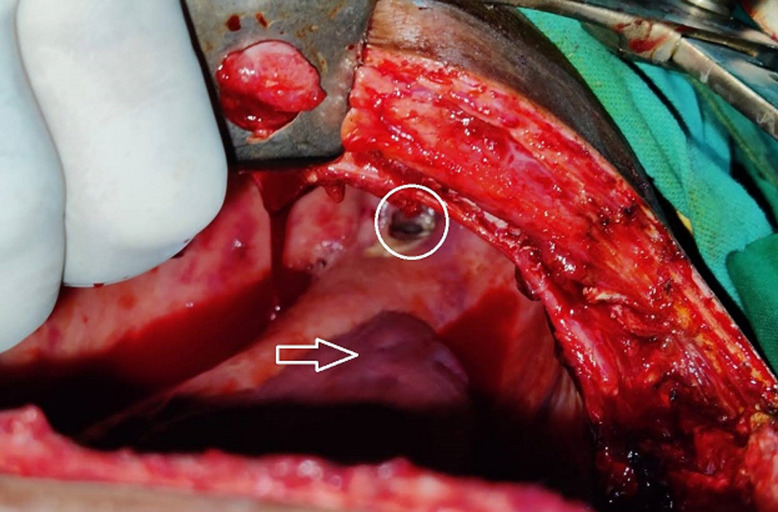
bronchial rent, white circle denotes the bronchial rent, white arrow showing the deflated lung

Surprise evidence of bronchial tear intraoperatively would be a nightmare for surgeon as well as anaesthetist (Figure 2). A high grade of clinical suspicion is needed for the tracheo-broncheal injury to be diagnosed, when the cause is not evident. In this case the whole surgical procedure of left lung decortication went uneventful till conclusion, when recruitment maneuvers were performed. There are many causative factors for tracheo-bronchial injury, which we would like to discuss as follows:

**DLT induced:** DLTs are the conventional choice for: a) isolated lung ventilation; b) single lung trauma; c) infection. In experienced hands, the placement of DLT is easy and complications rarely arise. However, the sizeable external tube and stiff stylet make the placement of a DLT risky. There are some case reports mentioning Carlen´s double lumen endotracheal tube (DLT)-induced trauma to tracheobronchial tree (incidence<0.2%) [2]. Apart from distal trachea, as there is an inclination to use left-sided DLTs, injuries were most frequently observed along the left main bronchus. These injuries commonly materialize as elongated tears in the trachea especially the membranous component [3]. Although rupture in the tracheobronchial tree occurs rarely, plausible risk factors include erroneous placement due to inexperience in managing airway, numerous intubation attempts, incorrect placement of the stylet through the DLT, over inflation of the cuff, insufficiently anesthetized patient, tracheomalacia secondary to chemotherapy, patients using steroids or undergoing radiation therapy, previous tracheal stenosis due to pulmonary tuberculosis [4].

Recognizing a tracheobronchial tear and timely repair are the pillars for a favourable outcome. The anesthetist might recognize untoward changes in vital signs, unanticipated challenges in ventilation, and the onset of a remarkable and unaccountable ventilatory leak. In this particular case, the tear was not initially perceived presumptively on account of ongoing successful ventilation and optimum left lung isolation. Due to uneventful proceedings of the procedure with no complications in ventilation or deterioration of vital signs, we did not suspect any untoward happenings. A substantial air leak after recruitment of left lung indicated by loss of tidal volume drew our attention to a potentially disrupted airway. A bronchoscopist can rule out the likelihood of a distal bronchial tear that was not discerned initially. Since we did not have a pediatric bronchoscope available in our institute, this could not be done. After examination of bronchial tear, surgeons could not see the DLT tip or blue colored balloon nearby. Hence the potential DLT trauma to left main bronchus is less likely. Manipulations of DLT may be required with changing position of patient for surgery, which was not required in our case.

**Surgical dissection induced:** tracheo-bronchial rent while doing major thoracic surgeries have also been reported in some case reports, especially whenever the surgical site is nearby main bronchus, for example hilar lymph nodes, carina, esophagus etc. [5]. In such scenario, abrupt changes in vitals are noted and the injured site has view of fresh lacerations. However, in the present case, the left lung was completely deflated and surgical site was away from bronchial rent which was around 2 x 2 cm circular shaped with scarred edges. Initial ventilator parameters did not show any loss of tidal volume and the leak was identified at the end of surgery, hence surgical trauma to left bronchus again seems less likely.

**ICD induced bronchial trauma:** penetrating trauma most commonly causes injury to the trachea, tracheal cartilage, or the ligamentous components between tracheal rings [6]. Most traumatic injuries to the tracheobronchial tree occur within 2.5 cm of the carina, of which mainstem bronchial injuries constitute a majority, exceeding 85% [7]. It is difficult to diagnose a bronchial injury as the earliest signs and symptoms such as dyspnea and subcutaneous emphysema are non-specific. However, a sizeable subcutaneous emphysema and a growing pneumothorax regardless of a chest drain can strongly point to a bronchial rupture. In our case the rupture was at the left main bronchus, which was not evidenced on computed tomography (CT), when reviewed preoperatively by the senior radiographer. It might be presumed that such a perceptible anatomical injury would be evident on imaging, steering the clinician into a false sense of security if imaging studies do not reveal any abnormality. However, a case report published in 2014 concluded that a bronchial rupture is not always evident on imaging or even on bronchoscopy [8]. If a substantial ventilator leak develops in a patient with chest trauma, even after many hours have passed since the injury, a high degree of suspicion is required. This did not happen in our patient with bilateral ventilation preoperatively.

For tracheobronchial rupture to be selectively treated, anastomosis through primary closure is required, which conserves lung and respiratory functions in 90% of cases [9,10]. Prompt extubation on table is recommended in such cases to avoid further injury by intermittent positive-pressure ventilation (IPPV). If elective ventilation is to be chosen, then airway pressures should be minimized and positive end-expiratory pressure should be provided.

## Conclusion

Whatever the cause of tracheo-bronchial rent during lung surgery, the importance of vigilant monitoring during perioperative period cannot be overemphasized. In present case, low exhaled tidal volume was noted in time and communicated with the surgical team. Adequate surgical repair and suitable ventilatory management form crucial steps in successful outcome of such complication.
